# Characteristics of the bitter and sweet honey from Algeria Mediterranean coast

**DOI:** 10.14202/vetworld.2019.551-557

**Published:** 2019-04-17

**Authors:** Ines Otmani, Cherif Abdennour, Amina Dridi, Labiba Kahalerras, Abdelaziz Halima-Salem

**Affiliations:** 1 Department of Biology, Laboratory of Animal Ecophysiology, Faculty of Sciences, University of Badji Mokhtar-Annaba, Annaba, Algeria; 2 Department of Biology, Laboratory of Plant Biology and Environment, Faculty of Sciences, University of Badji Mokhtar-Annaba, Annaba, Algeria; 3 Department of Pharmacy, Laboratory of Bromatology and Hydrology, Faculty of Medicine, University of Badji Mokhtar-Annaba, Annaba, Algeria

**Keywords:** antioxidant activity, bitter honey, flavonoids, polyphenols, sweet honey

## Abstract

**Aim:**

This study aimed to compare the physicochemical, the biochemical, and the antioxidant characteristics of unifloral bitter honey and polyfloral sweet honey.

**Materials and Methods:**

Unifloral bitter and polyfloral sweet honey samples were, respectively, harvested in January and July, and then, they were extracted by the traditional method. The markers of refractive index, pH, free acidity, Brix index, density, reducing sugars, total polyphenols, flavonoids, tannins, 2,2-diphenyl-1-picrylhydrazyl (DPPH), and ferric reducing/antioxidant power (FRAP) were evaluated.

**Results:**

The obtained results showed that the physicochemical parameters are within the normal ranges, in which they meet the international standards (Codex Alimentarius). For biochemical constituents, matching concentrations of reducing sugars (glucose+fructose) were observed in both samples, while that of sucrose were very low in unifloral than polyfloral honey. The levels of the active ingredients showed a difference in total polyphenols and tannins of the two types of honey studied, whereas that of flavonoids were almost similar. The antioxidant activity of various samples evaluated by DPPH and FRAP indicated that unifloral has a superior activity.

**Conclusion:**

Compared to polyfloral honey, unifloral bitter honey has lower sucrose, high total polyphenols, and tannins levels, in addition to higher antioxidant potential.

## Introduction

The honeybee has been known for its healing and nutritional benefits since antiquity. Honey is not only used for the treatment of human diseases, but also it is extended to domestic animals, as it is effective during infections and wound injuries in inhibiting the growth of fungi and bacteria, even in the case of microorganism resistance. Previously, researchers proved that the properties of honey make it as one of the important natural antioxidants [1,2]. In addition, it was reported that honey with higher water content, darker color, and high phenolic content has more antioxidant activity [3,4]. However, the physicochemical properties, the chemical composition, and the therapeutic applications were reported concerning honey from many parts of Algerian territory [5-9]. At this point, honey is known in traditional medicine as a preventive as well as a treating agent by boosting the immunity, fighting anemia, healing wounds, stabilizing heart function, activating digestion, curing skin allergy, and inhibiting pathogenic bacteria [2,3].

Honey chemical composition can be affected by the climatic factors and the plant species. The major nectar of polyfloral honey in Algeria is collected during springtime characterized by high plant diversity [6,7,10,11]. On the other hand, unifloral bitter honey is produced mainly from the nectar of the strawberry tree *Arbutus unedo*, a typical product of Mediterranean basin, in late autumn-early winter where the flowers of other plants are scarce. Thus, the physicochemical properties, the chemical composition, and the antioxidant activities of strawberry tree honey (STH) have given much attention in the northern Mediterranean region [12-15]. Indeed, the knowledge has been widened recently, in which it was suggested to be used as a chemopreventive agent due to its potentiality to inhibit colon cancer even at low concentration [15]. Despite that, little is known in terms of its physicochemical parameters and bioactive properties, at the local level, although bitter honey produced from the strawberry tree is a field of attraction among local population regarding its therapeutic advantages and respectable reputation.

This study aimed to compare the physicochemical, the biochemical, and the antioxidant characteristics of unifloral bitter honey and polyfloral sweet honey. Both kinds of honey were collected from Algeria Mediterranean coast.

## Materials and Methods

### Ethical approval

The Ethical Committee of Animal Sciences at the University of Badji Mokhtar-Annaba has given the authorization to realize the Ph.D. research program.

### Sample collections

Two types of honey were obtained from two unpolluted sites situated at Skikda Province, Northeastern Algeria, from experienced traditional beekeepers. Unifloral honey was obtained in January from Cap de Fer forest at El Marsa (H2) (Geographic coordinates: Latitude: 37.0299, Longitude: 7.25317; 37°1’48’’North, 7°15’11’’ East), where the available nectar was dominated by strawberry tree flowers (*A. unedo)* during November-December. Polyfloral ripen honey was collected in July from Bourzem countryside, daira of Sidi Mezghiche (H1) (Geographic coordinates: Latitude: 36.6833, Longitude: 6.71667; 36°40’60’’North, 6°43’0” East), having high biodiversity of flowery plants and remote from any type of pollution. The ripen honeys were extracted by the traditional method in which honey was removed from frames by centrifugal forces, filtered, placed in glass containers, and then stored in a dark place at room temperature.

### Physicochemical properties

The pH, refraction index, Brix concentration, density, and free acidity content were evaluated using the Harmonized Methods of the International Honey Commission [16].

### The pH

The pH was measured using a HI 9025-HANNA pH meter on a 10% honey solution in distilled water [17].

### Humidity

The humidity (water content) was obtained by the optical measurement of the refractive index of honey at 20°C by the use of correction coefficient 0.00023/°C. A drop of honey was taken by spatula, and then, it was deposited and spread thinly on the prism plate. Reading was made through the eyepiece at the horizontal dividing line between the light and the dark areas. The result of the refractive index obtained was compared to the Chataway table [16], which indicates the corresponding humidity in honey as a percentage.

### Total dry matter

Dry matter of honey was calculated as total soluble solids (TSS) and total solids (TS). The TSS was measured by an Exacta and Optech Labcenter Abbe Refractometer (Model RMT) and the results were expressed in Brix. All the measurements were done at ambient temperature and the readings were corrected for a standard temperature of 20°C by adding the correction factor of 0.00023/C [14]. The total soluble (TS) of honey sample was calculated as a percentage from the results of humidity using the following formula: TS (%)=100–humidity [18].

### Specific gravity

According to Nandaa *et al*. [19], the specific weight “density” of honey depended mainly on its water content. The test portion was 5 ml of honey and the same for the distilled water that was weighed. The density of honey was given by the following formula: d = m/m’.

### Free acidity

The acidity level was obtained by the titrimetric method. 10 g of sample was dissolved in 75 ml of carbon dioxide-free water in a 250 ml beaker. The solution was stirred and then titrated with 0.05 N sodium hydroxide (NaOH) (Chem-Lab NV, Belgium) to pH=8.3, in the presence of 4-6 drops of phenolphthalein until a persistent pink color appeared for 10 s. A blank test with distilled water was performed, and the volume of soda was corrected. Free acids and hydrogen ions were released by undissociated acids during titration [17]. The acidity level was expressed in mEq/kg honey.

### Determination of sugars

Reducing sugars (glucose and fructose) and sucrose were evaluated by the method of Luff-Schoorl [20], where sugars were extracted with water and then measured before and after inversion. Results were calculated according to the table of Luff-Schoorl which indicates the number of mg of sugars corresponding to (n2-n) and (n2-n1) ml of 0.1 N sodium thiosulfate pentahydrate (BIOCHEM, Chemopharma, Canada) per 25 ml of sugar solution, with n: Before inversion, n1: After inversion, n2: Control.

### Determination of active compounds

#### Total polyphenols

The estimation of total phenolic compounds was carried out by the Folin–Ciocalteu colorimetric method. The honey was diluted in distilled water (500 mg/ml). Then, 1.5 ml of the Folin–Ciocalteu reagent (CARLO ERBA, France) (diluted with 1 ml of the reagent in 10 ml of distilled water) and 1.2 ml of the sodium carbonate (Na_2_CO_3_) (Sigma-Aldrich, USA) (7.5%) were added to 0.3 ml of diluted honey. After incubation in the dark for 1 h, the absorbance was obtained at 760 nm. The concentration of total phenolic compounds was determined by reference to the calibration curve of gallic acid performed in parallel [21]. Results were expressed in mg of gallic acid (Biochem, Chemopharma, Canada) per 100 g of honey (mg GAE/100 g).

#### Total flavonoids

Total flavonoid content of honey was determined by a colorimetric method. 1 ml of honey was mixed with 4 ml of distilled water, and then, 0.3 ml of sodium nitrite (Biochem, Chemopharma, France) solution was added. After 6 min, 0.3 of aluminum chloride (Sigma-Aldrich, Germany) (10%) was added, the mixture was left standing for 6 min, and then, 2 ml of NaOH 1N (Chem–Lab NV, Belgium) solution was added, and the total was adjusted to 10 ml with distilled water. The mixture was allowed to stand for 15 min, and then, the absorbance was measured relative to blank at 510 nm [22]. Results were expressed relative to catechin (mg catechin/g dry matter).

#### Total tannins

Total tannins were measured by the method of Folin-Denis [23]. The colorimetry of the tannins was based on the measurement of the blue color formed by the reduction of phospho tungsto molybdic acid by tannins like compound in an alkaline medium. 1.0 ml of extract and a standard solution of tannic acid (100-800 µg/ml) were made up to 7.5 ml with distilled water. Then, 0.5 ml Folin-Denis reagent (Sigma-Aldrich, USA) and 1 ml of Na_2_CO_3_ (Sigma-Aldrich, USA) solution were added. The volume was made up to 10 ml with distilled water, and then, the absorbance was read at 700 nm. The total tannin content was expressed as mg of tannic acid equivalent/g extract [24].

### Determination of antioxidant activities

#### Free radical scavenging activity (DPPH assay)

The antioxidant properties of the honey sample were studied by evaluating the free radical scavenging activity of the 2,2-diphenyl-1-picrylhydrazyl (DPPH) radical, which was based on the method of Boulilaa *et al*. [25]. The DPPH radical was one of the most commonly used substrates for rapid and direct assessment of antioxidant activity due to its radical-shaped stability and simplicity of analysis [26]. Inhibitory concentration (IC_50_) was defined as the concentration of honey that inhibits 50% of the DPPH radical. The lower value of the IC_50_ (substrate concentration that causes 50% inhibition of DPPH activity) indicated higher antioxidant activity. 1 ml of each honey extract at different concentrations was mixed with 2 ml of a methanolic solution of DPPH at 0.04 g/l. After 60 min incubation in the dark at room temperature, the absorbance was measured at 517 nm using methanol as a blank. The DPPH inhibition percentages were calculated according to the following formula: DPPH (% inhibition)=(A_0_-Aeq)/A0×100, where A_0_ and Aeq are the absorbance of the control and the sample, respectively. The curve expressing the percentage inhibition of DPPH as a function of the concentration of the antioxidant (μg/ml) made it possible to deduce the IC_50_ defined as the concentration of antioxidant necessary to decrease the initial concentration of the DPPH at 50%.

#### Ferric reducing/antioxidant power assay (FRAP assay)

The FRAP assay was based on the ability of antioxidants to reduce ferric iron Fe3^+^ to ferrous iron Fe2^+^. FRAP assay was measured according to the method of Oyaizu [27]. After dilution with distilled water, 1 ml of each honey sample was mixed with 2.5 ml of a phosphate buffer solution (0.2 M, pH 6.6) and 2.5 ml of potassium ferricyanide at 1% (m/v). The mixture was then incubated at 50°C for 20 min. 2.5 ml of 10% (w/v) trichloroacetic acid was then added, and the mixture was centrifuged at 3000 rpm for 10 min. To 2.5 ml of each supernatant, 2.5 ml of distilled water and 0.5 ml of 0.1% (w/v) ferric chloride were added. The absorbance was measured at 700 nm against methanol as a blank. Ascorbic acid was used as a positive control. The result was expressed by the effective concentration (EC_50_) of the aqueous extract of honey corresponding to an absorbance of 0.5 µg of honey/ml of the mixture (EC_50_ in mg/ml).

### Statistical analysis

Statistical analysis of all results was analyzed using the Microsoft Excel program (2010) and “Minitab 17” software (Student’s t-test). Experiments were prepared in triplicate, where the mean ± standard deviation was calculated.

## Results

### Physicochemical properties and sugar levels

Results in Table-1 show the characterization of honey according to their physicochemical properties. The two types of honey are close in their properties, except for bitter honey that has low sucrose level (0.95 g/100 g) compared to sweet honey (5.89 g/100 g).

**Table-1:** Physicochemical properties and concentration of reducing sugars (glucose and fructose) and sucrose of sweet and bitter honey.

Parameters	Units	Sweet honey	Bitter honey
pH		4.30	4.59
Refractive index		1.4915	1.4845
Humidity	%	18	20.8
TSS	Brix	80.5	77.7
TS	%	80	79.2
Specific gravity		1.38	1.40
Free acidity	mEq/kg	41	37
Reducing sugars	g/100 g	60.4	60.4
Sucrose	g/100 g	5.89	0.95

TSS=Total soluble solids, TS=Total solids

### Bioactive compounds

**Figure-1:**
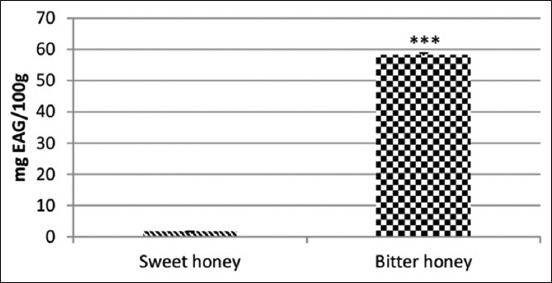
Total phenolic content of polyfloral sweet and unifloral bitter honey. mg GAE/100 g honey: mg Gallic acid equivalent/100 g honey.

**Figure-2:**
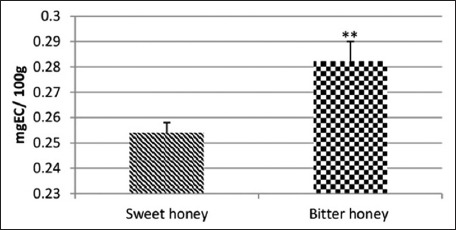
Total flavonoid content of polyfloral sweet and unifloral bitter honey. mg EC/100 g honey: mg equivalent catechin/100 g honey.

**Figure-3:**
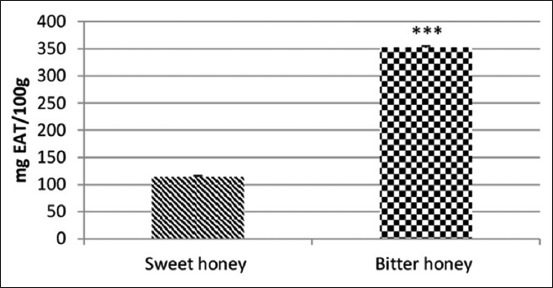
Tannin content of polyfloral sweet and unifloral bitter honey. mg ETA/100 g honey:mg equivalent tannic acid/100 g honey.

Results indicate that bitter honey had a remarkable raised amount of total polyphenols, flavonoids, and tannins than that of sweet honey (Figures-1-3).

### Antioxidant activity

Table-2 shows that bitter honey had a greater antiradical and antioxidant activity (DPPH and FRAP) than sweet honey. The IC_50_ of DPPH was 42.74 mg/ml ascorbic acid for sweet honey, but it was 5.72 mg/ml ascorbic acid for that of bitter honey. Moreover, the FRAP test recorded wide differences in IC_50_ between the two kinds of honey.

**Table-2:** The antioxidant activity (DPPH and the FRAP assay) of sweet and bitter honeys.

Parameters	Units	Sweet honey	Bitter honey
DPPH (IC_50_)	mg/ml	42.74±1.63	5.72±0.33
FRAP (IC_50_)	mg/ml	106.38±0.82	34.91±1.40
Ascorbic acid	mg/ml	3.20±0.10	2.23±0.13

DPPH=2,2-Diphenyl-1-picrylhydrazyl, FRAP=Ferric reducing/antioxidant power, IC_50_=Inhibitory concentration

## Discussion

### Physicochemical properties and sugar levels

Compared to the bitter honey, sweet honey has lower pH, humidity, and specific gravity, but it has higher TSS, TS, and free acidity. The characteristics of sweet and bitter honey collected from sites with close climatic conditions have revealed some interesting differences that were related to their chemical composition. Subsequently, it is a reasonable expectation that the composition and properties of honey from various locations might differ [28]. In addition, bitter honey was darker to some extent.

The refractive index of the samples studied is between 18% for unifloral and 20.8% for polyfloral (Table-1). The lowest value of unifloral is well below the limit (norms: Max. 21%) recommended by Codex [17], while that of polyfloral is slightly above the recommended values of 20%. Moisture content is an important element in assessing the degree of honey maturity and its shelf life. In general, a high amount of water causes the fermentation of honey, loss of flavor and quality. The fermentation of honey during storage is caused by the action of yeast osmotolerance, leading to the formation of ethyl alcohol and carbon dioxide. The used honey is remote from these conditions since samples were analyzed after a short period of collection. On the other hand, the bitter taste of honey from site H2 is definitely due to the presence of nectars mainly from *A. unedo*, followed by *Inula viscosa* plants because their flowering period is in late autumn and earlier winter. The refractive index of multifloral, *Eucalyptus* and *Citrus*, collected from different regions of Algeria had a water content of 13.9-20.2% [6] and 16-20.4% [5], respectively. Yet, the moisture content of STH was 18.6% and 18.9% for the Portuguese and the Italian, respectively [12,29]. The relatively higher moisture content of bitter honey in this study is probably due to the cold-humid season of production.

The pH values of the two samples analyzed (Table-1) are consistent with those reported by Bogdanov *et al*. [30], who confirmed that honey derived from nectar had a pH of 3.5 to 4.5, while those from honeydew were between 5 and 5.5. Notably, the pH values of Algerian multifloral honey were 3.69-4.48 [11], which looks within the range of the actual investigation. Notably, honey was extracted by the traditional method using hand extractor, and then, it stored in optimal conditions. The pH of the STH honey seems higher, where samples from Portugal showed a mean value of 4.52 [13] and 4.49 [31], while that from Italy had a pH of 4.2 [29].

The value of the free acidity (Table-1) is in the standards of 50 mEq/kg set by the Codex [17]. Hence, acidity is an important criterion of quality; it gives very important indications of honey status [32]. In addition to the organic acids and amino acids, the botanical diversity plays an important role in defining honey final pH. Previously reported, the free acidity of Algeria honey was 47.25-55.25 [33] and that of Moroccan Carob honey varied between 17 and 42 mEq/kg [34]. Italian unifloral STH showed levels of 27.3-53.4 mEq/kg [29] that is near to the actual study.

TSS of honey that represents the dry material mainly sugar compounds was 80.5° for unifloral and 77.7° for polyfloral (Table-1). TS had close values in which it was 80% for sweet honey and 79.2% for bitter honey. The obtained results are in total agreement with the literature cited [35]. Moreover, the mean levels of TSS (76.2-80.40) from India honey [18] and that of TS (l 79.0-82.2%) from Portugal agreed well with our results for both types of honey. Accordingly, the TSS content of STH samples from south Portugal showed a mean level of 78.35 Brix [13].

The specific gravity of sweet and bitter honey is, respectively, 1.38 and 1.40 (Table-1); they are more or less in the normal range. In Western and Southwestern Algeria, the specific gravity of honey was 1.405-1.442 [33] and for Northeast region was 1.32-1.55 [36]. Accordingly, the specific gravity of Indian honey (1.39-1.49) is almost similar to our results [19]. Notably, it seems that honey samples having high moisture content had the lowest specific gravity and vice versa.

The reducing sugar levels of the current study are 60.4 g/100 g for both types of honey (Table-1). According to the Codex [17], total and reducing sugar content must not be <60 g/100 g of flowery honey, which confirms that the investigated samples are nectar honey. Furthermore, honey from different regions of Northeast Algeria had reduced sugars of 62.5-84.45%, and sucrose was 2.91-15.34%, whereas that of unifloral honey (*Ziziphus jujuba*) was 62.5-90.12% for reduced sugars and 4.24-10.09% for sucrose [8]. It was reported that fructose, glucose, and sucrose level of Finnish and sweden polyfloral honey was 38.7-43.7, 30.4-38.1, and 0.0 g/100 g, respectively [37]. It looks that the percentage of carbohydrate of sweet honey from this study is close to the Nigerian and Indian results [38,39], but sucrose is much lower in bitter honey with a concentration of only 0.95 g/100 g. For the STH bitter honey, reducing sugars content of this work is lower than that reported from Portugal [13] and Italy [29].

### Bioactive compounds

The unifloral bitter honey is richer in the total polyphenols than that of sweeter one, suggesting of having better antioxidant potential. Phenolic substances are the main factors responsible for the biological activities of honey. Pakistani honey had a mean of 48.5 mg/100 g honey [40], while Chili honey recorded levels of 0-8.83 mg/100 g honey [41], which is close to our unifloral honey. Interestingly, Italian STH was the richest in total phenols (972 mg/kg GAE) compared with honeydew, heather, *Eucalyptus*, asphodel, *Citrus* spp., and *Acacia* ones [14]. Seemingly, our results agree well with that of Portuguese STH [12], but not with the STH from the different regions of Sardinia, which recorded very low total polyphenols content [15]. Remarkably, homogentisic acid is the main phenolic compound of STH, which might provide an important antioxidant potential. In this study, honey is made mainly from the flowers of strawberry tree *A. unedo*, but the plant *I. viscosa* participates to some extent, in addition to other rare flowers.

The content of flavonoids in both types of honey is almost the same, despite the difference in color, where the color of bitter samples is darker. In general, darker honey such as sunflower and buckwheat contains higher concentrations of flavonoids than bland honey, as well as greater antioxidant capacity [33]. Further, higher values of flavonoids were recorded in Algerian [42], Pakistani [40], and Malaysian honey [43]. However, Chili honey is within the range of our results, in which it contained between 0.01 and 8.83 mg/100 g honey [41]. The total flavonoids content of STH (65.74-108.20 mg CAE/kg honey) from Sardinia [15] is closely related to our results of unifloral honey, while that from Portugal (4.09-5.77 mg QE/100 g honey) was remarkably lower [13].

The content of tannins has recorded the highest level in bitter honey that is in agreement with the dark color observed. Tannins are phenolic polymers consisting of multiple anthocyanin-like molecules [44]. Indeed, the characteristics of woody aroma and dark color of honey indicate the presence of tannins and lignins. Moreover, polyfloral honey from Benin had values much higher than our polyfloral and unifloral honey [45]. For TSH, it was reported to be more active as an antioxidant agent due to the higher content of tannins [38]; this is likely why the unifloral bitter honey is more appropriate for therapeutic purposes.

### Antioxidant activity

The DPPH assay provided an IC_50_ of 42.74 and 5.72 mg/ml ascorbic acid for sweet and bitter honey, respectively (Table-2). The lowest inhibition value indicates a strong ability to trap free radicals [46]. As a result, the DPPH assay was reported to reflect the activity of water-soluble antioxidant. It seems that the DPPH of two unifloral Algerian honey from the southern region had relatively strong values of 10.94 and 6.60 IC_50_ mg/ml honey [47]. Accordingly, other studies revealed that STH of the coastal region of Italy [14] and Croatia [48] also showed higher DPPH activity of, respectively, 4.8 mmol TEAC/kg and 3.34 mmol TEAC/kg, which agree well with this study. Differently, the DPPH of STH has documented very strong activity (0.09-0.20 mmol TE/100 g) from different regions of Sardinia [15].

The FRAP assay has given an IC_50_ of 106.38 for sweet honey, while it reached 34.91 mg/ml ascorbic acid for bitter honey (Table-2), suggesting a dissimilar antioxidant potential. The antioxidant potential is attributed mainly to the presence of different phenolic components such as flavonoids, phenolic acids, and other compounds that have different antioxidant potentials. In addition, light-colored honey had lower FRAP values than dark-colored one, indicating a strong antioxidant capacity. In Portugal, the reducing power of honey varied between 13.26 and 94.11 mg/ml, depending on honey color [49], a result appears to be similar to the present investigation. For Maltese honey, a significant correlation between the antioxidant activity and total polyphenol was recorded [50]. Opposing to our results, the FRAP activity of Italian STH was higher (11.7±1.7 mmol Fe2^+^/kg) than the other unifloral honey such as *Eucalyptus*, *Citrus*, and *Acacia* [14]. On the other hand, Sardinian bitter honey had low FRAP activity of 0.51-0.92 mmol Fe(II)/100 g [15]. Overall, the antioxidant activity noted provides the honey, especially the bitter one with the appropriate power to counteract the oxidative stress generated within cells when exposed to different diseases and toxic agents. Such results confirm some popular undocumented views about the application of STH in treating many diseases such as cancer and digestive disorders.

## Conclusion

In this study, the physicochemical characteristics, biochemical composition, and antioxidant activity of the unifloral bitter honey were investigated and then compared to the polyfloral honey. Both kinds of honey have close physicochemical properties with good quality. Concerning the active compounds, bitter honey contained higher amounts of total polyphenols and tannins, reflected in their strong antioxidant potential. The discussed results indicated that the investigated honey has close characteristics of honey samples reported elsewhere, especially for the Mediterranean basin.

## Authors’ Contributions

IO managed the experimental work and wrote the manuscript, CA collected the samples and corrected the manuscript in English language, AD did the assays of the bioactive compounds and the antioxidant activities, LK participated in all the assays realized, and AH carried out the physicochemical study. All authors read and approved the final manuscript.
